# Surfing Alone: From Internet Addiction to the Era of Smartphone Dependence

**DOI:** 10.3390/ijerph21040436

**Published:** 2024-04-03

**Authors:** Amelia Rizzo, Dario Alparone

**Affiliations:** 1 Department of Clinical and Experimental Medicine, University of Messina, 98122 Messina, Italy; 2 Department of Cognitive Sciences, Psychological, Educational, and Cultural Studies, University of Messina, 98122 Messina, Italy; 3 Department of Psychology, Faculty of Letters and Human Sciences, University of Western Brittany, 20 rue Duquesne, CEDEX 3, 29238 Brest, France; dario.alparone@univ-brest.fr

## 1. Introduction

Technological addiction refers to behavior characterized by excessive and prolonged use of technological devices; reactions of anger, frustration, or panic when unable to use them; and unsuccessful attempts to reduce the time spent on them. The first attempts to define addiction date back to Kimberly Young [1], who focused on Internet addiction, defining it as a “controversial new addiction”. As described in her book “Caught in the Net”, she launched one of the first online research studies by posting questions—which later formed the items of the Internet Addiction Test (IAT)—to network users. She received almost 500 responses, with 80% showing an addicted profile. These users also described relational and adjustment issues stemming from excessive Internet use, namely a compromise in social and work functioning.

Subsequently, Davis [2] felt the need to define a cognitive–behavioral theoretical model of Problematic Internet Use (PIU), focusing on maladaptive cognition associated with PIU rather than on behavioral factors. Particularly, the author distinguished between specific PIU (pathological use of the Internet for a particular purpose, such as online sex or gambling) and generalized PIU (a broader set of behaviors). Almost simultaneously, Shapira and colleagues [3] were working on a more inclusive diagnostic framework, arguing that definitions based solely on addiction or pathological gambling were not inclusive enough to capture the characteristics of the PIU user population and could create clinical and psychodiagnostic problems.

**Figure 1:**
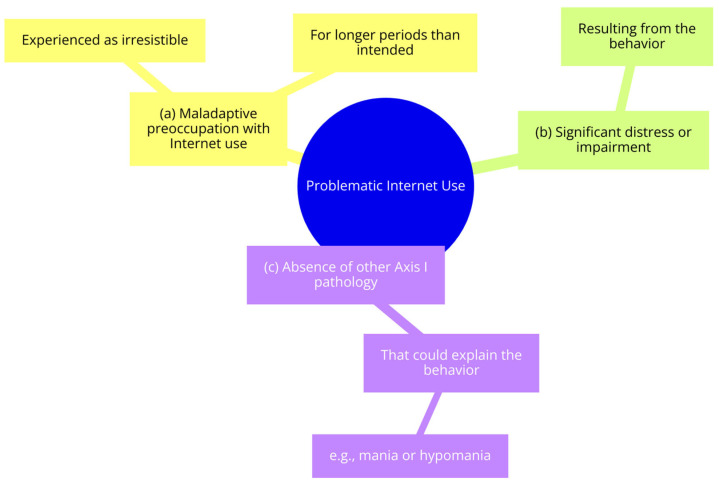
Problematic Internet Use Model.

Over time, the label “Internet Addiction”, due to its partial correspondence with pure addiction, was abandoned in favor of the “Problematic Internet Use” model (See Figure 1), defined with the following criteria:

(a) Maladaptive preoccupation with Internet use, experienced as irresistible for longer periods than intended;
(b) Significant distress or impairment resulting from the behavior;
(c) The absence of other Axis I pathology that could explain the behavior, such as mania or hypomania [4].

In subsequent years, studies on Internet addiction and social networks have grown exponentially. The scientific literature began to focus especially on the explosion of Facebook, founded by Mark Zuckerberg in 2004. Particularly in clinical and social psychology, the focus shifted to the relationship between personality and the use of social networks or variables such as anxiety, depression, social anxiety, the ability to establish bonds, and pathological dependence. One of the most interesting studies investigated the underlying motivations for Facebook use [5] also in an Italian sample [6], identifying, to some extent, the psychological needs that it satisfies or seeks to satisfy, summarized in seven main components. According to Joinson’s 2008 study, Facebook use and motivation can be summarized in 7 factors: (1) Social Connection; (2) Social investigation; (3) Share Identities; (4) Social Network surfing; (5) Status update; (6) Contents; (7) Photographs.

It is interesting to note how the authors speak of use rather than abuse. In those years, the diffusion of SNS was so explosive that millions of individuals in a short period began to use an online profile: a digital version of themselves where they could rediscover lost contacts, strengthen new bonds, or establish connections with people they do not know in reality. Given the above criteria, it is clear how this could lead some individuals to develop addictive behavior, as “being on Facebook” implied spending a large amount of time, losing track of time, and difficulty reducing its use. Moreover, news reports showed an increase in divorce sentences due to Facebook, with repercussions in the relational and family spheres. All the characteristics of what was defined as Facebook Addiction or Facebook Addiction Disorder seemed to be configured. However, attempts to introduce these terminologies or actual diagnostic criteria in the statistical manuals on mental disorders failed.

The scientific literature showed rather contrasting data, and in the meantime, the spread of the Internet, social networks, online games, and especially smartphones became more and more natural in our routines. The boundary between use and abuse became increasingly blurred, appearing rather as a continuum with different degrees of functioning and/or impairment.

The scientific debate on Problematic Internet Use began to focus on individual vulnerability. Starting from the assumption that the Internet represents an excellent resource to escape daily worries and isolate oneself in an idyllic reality, it was hypothesized that there are subjects particularly exposed to the risk of developing a dependence on the Network, presenting some common aspects: existential problems, low self-esteem, difficulties in social relationships, a tendency to isolate.

## 2. A Multicomponent Model of Problematic Internet Use

Considering these aspects of vulnerability, Cantelmi and Talli [7] proposed a model in which the subject and their self-perception, how the user perceives reality, and how the user perceives the virtual world are considered. Depending on the quality of representation (good/bad), the authors hypothesized four fundamental positions, distinguishing between specific PIU (thus focused on a specific function of the Internet such as erotic material, gambling, and auctions) and generalized PIU (referring to a generalized and multicomponent overuse of the Internet, often associated with abuse of chat and email, for example).

In the first possible condition, the subject uses the Network as an expression of autistic withdrawal, to not think about themselves or the surrounding world. Probably, the PIU is generalized; reality is perceived as bad and hostile, the subject perceives themselves as bad, while virtuality is represented as good.

In a second configuration, the subject uses the network as a means of escape or flight from an unsatisfactory reality; in this case, the PIU is potentially generalized, and reality is, thus, perceived negatively, while the subject and virtual reality are good objects.

A third possible configuration is where the subject perceives both reality and virtuality as good and themselves as bad. The use of the network is, thus, motivated by the need to increase self-esteem (self-care) or as a mediator/facilitator of their relations with reality, for which the PIU is plausibly generalized.

Finally, there can be a case where the subject perceives reality as good, as well as themselves and virtuality. In this case, they use the network to increase their level of excitement or to achieve certain goals, and this often manifests with a more specific PIU.

The only case in which a problematic relationship cannot be configured is where the subject perceives themselves, reality as simultaneously good and bad, and virtuality as bad. However, thinking about today’s world, it is no longer possible to consider virtual reality only as a threat. In the author’s opinion, virtual reality should also be considered as potentially both good and bad. Only this process indicates a low degree of splitting and a high level of integration, as we will see later.

We must remember that scientific literature on network use shows experimental results in stark opposition, probably also the result of a split view and studies that seek to demonstrate harmful effects rather than beneficial ones, reflecting the theoretical beliefs of the experimenters. For a long time, indeed, there has been talk of the damages that the use of the Internet or social networks or video games can cause to the mind, with particular concern to the condition of the adolescent, in a delicate and extremely plastic hormonal, biological, and psychic phase, which can also be defined as physiologically vulnerable. In this phase, from the ashes of pre-existing structures—the infantile mechanisms so far functioning, put in crisis by the environment and the demands of the external and internal world—the Ego must arise through the definition of identity and the discovery of oneself and one’s personal traits.

So, how much can the Internet and online video games interfere with this identity structuring process? And to what extent are Internet addiction and pathological abuse of the network responsible for psychopathological drifts?

## 3. The Problematic Use of the Internet: A Primary or Secondary Disorder?

In the literature, however, the relationship between Problematic Internet Use (PIU) and psychotic disorders has been somewhat neglected. Gibbs [8] emphasized that various types of addiction and heavy Internet use are predictable in patients with specific personality traits, such as narcissists, who use the web as an essentially self-related object, thus serving self-esteem and the need for admiration; the depressed or avoidant, who seek to avoid the risk of relational loss by controlling others through a relationship confined to the virtual world; the ‘schizoid or paranoid’ who retreat into the cyber world to escape from a persecutory or nonexistent reality, preferring a safe inner world to the catastrophic fears that come from the real world. Despite these theories, there are very few empirical and longitudinal empirical studies dealing with the evolution of Problematic Internet Use in psychotic paths. Specifically, we know from Mittal, Dean, and Pelletier [9] that individuals experiencing psychotic-like experiences (PLEs) can be divided into two subgroups with different outcomes. In the first group, where psychotic-like experiences improved, there was a decrease in Internet use, while in the second group, where psychotic-like experiences worsened, there was a significant increase in the intensity of Internet use as a substitute for reality at two months. But is it the use of the Internet that induces similar psychotic states or worsens similar psychotic symptoms, or is it rather a premorbid state manifesting itself with pathological addiction to the Internet? A single case study [10] published in the journal *Computers in Human Behavior* examined precisely whether Problematic Internet Use could evolve into a pre-psychotic state or vice versa. The case, diagnosed as a pre-psychotic state, had a history of 4 years of progressive, symptomatic worsening, with relational closure and severe cyber addiction behaviors. The withdrawal corresponded to a high amount of time spent online in an MMOG (Massive Multiplayer Online Game) in the role of an armed policeman. The symptoms were characterized by fears and worries about fantastical and monstrous characters. Moreover, there was a condition of school dropout, with significant consequent impairment of social functioning. Two different explanatory hypotheses were advanced.

Excessive and prolonged use of the Internet may have led to an almost psychotic condition. In other words, the Problem of Internet Use (PIU) manifested first, meeting the three diagnostic criteria defined by Shapira and colleagues in 2003 [4]. Over the years, maintaining dysfunctional behaviors such as procrastination and the experience of immersion in the online world as described by Thatcher, Wretschko, and Fridjhon in 2008 [11], the pre-psychotic condition emerged with its abnormal and worrying characteristics linked to the excessive use of video games and the creation of disturbing images of fantastical and monstrous entities.

Other research identified isolated cases where it seemed that Internet use generated delusional phenomena [12]. However, these authors suggested that media content can integrate into delusional thoughts, indicating that such phenomena might be more related to other psychiatric pathologies rather than being a disorder in itself.

In addition, according to available longitudinal studies, there is no evidence that PIU can evolve into a pre-psychotic condition. Even Mittal and colleagues, in 2013 [9], examined PIU in subjects with previous psychotic experiences, suggesting that a cause–effect relationship is not appropriate in this context.

Thus, the problematic use of the Internet might be more a manifestation of a broader psychopathological disorder rather than an independent disorder.

In the clinical case examined the use of the Internet could be interpreted as an attempt to adapt to a reality experienced as challenging. This emerged from the analysis of the results of the Wartegg projection test, conducted following the method of Alessandro Crisi, head of the Italian Wartegg Institute [13]. During this test, complex patterns were detected. It seems that Roberto integrated various mechanical elements (such as traffic lights, clocks, and gaming consoles) into the symbolic representations linked to his identity, sexuality, and father figure. Both in his behavior and in his deep thoughts, virtual reality was used as a sort of substitute for physical reality, a positive and accessible element to cope with disorganized attachment.

Griffiths [14] suggested that, in most cases, excessive use of the Internet reflects behaviors that provide satisfaction, whereas the network is primarily used to facilitate such behaviors. Consequently, the Internet would act more as a tool rather than as a direct cause of other disorders, as stated by Shaffer, Hall, and Bilt in 2000 [15]. Internet addiction, therefore, could be more an indicator of psychological distress, particularly evident in predisposed individuals, who are more prone both to problematic computer use and to other psychiatric conditions, as observed by Yellowlees and Marks in 2007 [16]. Regarding the field of research, the examined case suggests that, although the diagnostic criteria for PIU have been effective in describing the patient’s addictive behavior, in light of the clinical history, they are not sufficient to corroborate the hypothesis of a disease, although some authors argue that so-called Internet should be included as a distinct diagnosis in the Diagnostic and Statistical Manual of Mental Disorders [17].

## 4. Dependency, Isolation, and Loneliness: A Clinical Perspective

In the clinical context, the case study emphasizes the significance of investigating computer use, especially among younger patients. This is due to the prevalence of computer addiction, notably among MMO (Massively Multiplayer Online) game players [18]. This addiction is quite common during adolescence, occurring in one out of five teenagers between the ages of 12 and 16 [19]. The study also indicates that the Internet might serve as a tool for defense from reality, particularly within the psychopathological spectrum of a psychotic nature. Addiction is known to correlate with certain personality aspects such as shyness [20], difficulties in social interactions and peer relationships [21], as well as depression and loneliness [22].

Loneliness is a distressing sensation stemming from a discrepancy between actual and desired social connections [23]. Many adolescents aged between 12 and 15 years report experiencing loneliness, with approximately 12–20% feeling lonely at least “sometimes” [24]. Feelings of loneliness can remain stable and become chronic from adolescence onwards. From a developmental perspective, loneliness can be associated with a range of negative outcomes in adolescents, including poor physical and mental health.

Measures to contain the spread of COVID-19, such as school closures, led to a nearly 100% reduction in young people’s social contacts outside their families. This significantly contracted their social experiences, likely increasing feelings of loneliness and potentially exacerbating psychological distress [25,26,27,28]. International studies conducted in various countries highlighted the association between increased loneliness and distress in the pandemic context. For instance, about a third of Spanish and Italian parents of children aged 3 to 18 years reported their child feeling lonelier following the introduction of quarantine measures [29]. Many parents also reported their children being more nervous, anxious, angry, and exhibiting mood swings. The containment measures might have had even more negative effects on adolescents than on adults or children, as many key developmental tasks of adolescence are achieved through interactions with others [30]. As adolescents move towards independence, they spend less time with family and more with friends and partners [31]. A recent study by Cooper et al. [32] examined the effect of loneliness, social contact, and parent–adolescent relationships on adolescents’ mental health during COVID-19 isolation in the United Kingdom. Approximately 890 adolescents aged between 11 and 16 completed questionnaires on loneliness, social contact, parent–adolescent relationships, and mental health difficulties during the first 11 weeks of lockdown, and about half completed a follow-up one month later.

It emerged that adolescents experiencing greater feelings of loneliness during isolation had poorer mental health quality of life in the initial assessment, yet loneliness did not predict greater mental health difficulties one month later. Additionally, among adolescents, those with closer relationships with their parents reported significantly less severe symptoms of mental health difficulties and lower levels of loneliness. Parental closeness was significantly associated with less psychological stress, even at the follow-up.

Clearly emerging is the notion that loneliness can be a state that is either transient or chronic and that there are factors that can be considered protective. It is important to distinguish between a state of desired loneliness, such as proactive isolation aimed at a goal (e.g., relaxation, taking time off, reading, self-care), and a state of enforced loneliness, as was the case with forced isolation due to extreme confinement and social distancing measures.

The relationship between Internet addiction, loneliness, and the effects of the pandemic is the subject of ongoing studies, as the effects will be observable in the future. However, tracing the history of the conceptualization of Problematic Internet Use, it is evident that new categories are needed with increasing speed and fluidity—as Bauman would argue [33]—to approach the understanding of the phenomena we are experiencing. Currently, the term ‘techno-addiction’ is used to refer to a set of behaviors related to the use of digital devices. It is undeniable that technology had already pervaded our lives before 2020, but the pandemic event certainly further increased overexposure to digital devices, social media, and the Internet. Millions of workers moved—where possible—their activities to remote working, and many professionals and businesses launched platforms to continue producing in different forms, reaching customers and patients worldwide. At the same time, millions of students from kindergarten to university have become familiar with distance learning for quite an extended period, familiarizing themselves with new or already known platforms, such as Teams.

## 5. From Internet Addiction to Smartphone Addiction

The criterion of time spent online diminished in significance as a measure of addiction. In our daily experience, it is common to keep smartphones on even at night, to be always connected, and to return home if forgotten, as it has become inconceivable to face a day without a mobile phone.

**Figure 2:**
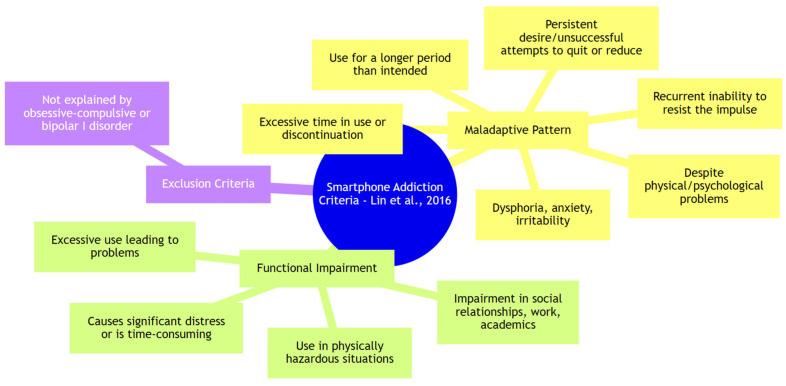
Smartphone addiction criteria [34].

Consequently, a new category emerged, proposed by Lin and colleagues [34] as early as 2016: Smartphone Addiction (see Figure 2). This refers to the dependency on a digital device that allows 24 h Internet access, fueling the need to be constantly connected. This addiction is not solely based on the amount of time spent online but also on the psychological and behavioral aspects associated with smartphone use.

Smartphone addiction encompasses various dimensions, including the compulsive checking of messages and notifications, the inability to limit usage time, withdrawal symptoms when not using the device, and the use of the smartphone to escape from problems or regulate mood.

This phenomenon reflects the broader impact of digital technology on everyday life, where smartphones have become integral to social interactions, information access, and even professional activities. The ubiquitous nature of smartphones makes them more than just a tool for communication; they are now a central part of personal identity and social existence. As with any form of addiction, smartphone addiction can have detrimental effects on mental and physical health, social relationships, and overall well-being. It is crucial to recognize and address this growing issue, especially considering the increasing reliance on digital technology in various aspects of life.

According to the proposal of these researchers from Taiwan, at least three of the criteria describing maladaptive behavior and significant impairment of social or academic functioning must be presented to diagnose smartphone addiction. Therefore, not only the time spent online but also attempts to resist use, time spent trying to reduce smartphone use, and withdrawal reactions play a crucial role in this model.

However, it should be noted that these criteria constitute a diagnostic proposal that, like its predecessors such as Internet addiction, Facebook Addiction, and Problematic Internet Use described in this chapter, has not been considered for inclusion in diagnostic manuals, such as the DSM-V [35].

## 6. Conclusions

Not only adolescents but all of us are immersed in the Network. The concepts of addiction to it, as we have seen, have seen many attempts to describe pathological situations and discriminate them from healthy use [36,37,38,39,40,41]. However, clinical observations on the events we are experiencing, on the acceleration of technological development, digital natives, the evolution of connection potential, and lastly, the pandemic with smart working and distance learning push for both theoretical and practical reflections [42,43,44,45,46,47].

In conclusion, it is observable how the models of conceptualization of Internet addiction, social media, Problematic Internet Use, and finally, smartphone addiction never achieved diagnostic independence. One must ask whether they are not the apex of deeper issues, the tip of an iceberg. In pathological situations, they often consist of the reason for seeking help or parents’ concern. But in everyday life, they raise issues of a relational, developmental, and public health nature, which deserve all the attention of studies and clinical practice.
